# Health Impact Assessment: Improving Its Effectiveness in the Enhancement of Health and Well-Being

**DOI:** 10.3390/ijerph120403847

**Published:** 2015-04-03

**Authors:** Jeffery Spickett, Dianne Katscherian, Helen Brown, Krassi Rumchev

**Affiliations:** School of Public Health, Curtin University, Perth Western Australia 6102, Australia; E-Mails: Dianne.Katscherian@curtin.edu.au (D.K.); H.Brown@curtin.edu.au (H.B.); K.Rumchev@curtin.edu.au (K.R.)

Most countries in the world have Environmental Impact Assessment (EIA) processes and procedures to evaluate the potential impact of development projects on the environment. This process, which attempts to predict the potential adverse effects of the proposed development project on the environment, is normally legislated and is part of the approval process for the project. Although these processes have been effective in reducing the adverse impacts on the environment they have been limited in their ability to provide protection for the health and well-being of people affected by the development. To bring attention to this limitation, the World Health Organisation (WHO) set out a series of principles for the better consideration of health and well-being impacts on human health [1]. Subsequent to this, a meeting resulted in the Gothenberg consensus, which identified Health Impact Assessment as a process specifically designed to evaluate both the negative and positive impacts of development projects on the health and well being of the potentially affected community [2]. Studies in the 1980s and 1990s had provided a better understanding of the determinants of health and the relevance of covering more than the physical environmental determinants to include economic, availability and access, social influences, lifestyle, personal, and biological factors [3]. These determinants provide a basis for a full consideration of the positive and negative impacts of not only development projects but also policies and programs on the health and well-being of the affected community including vulnerable sections of the community and issues of equity.

The impact of development projects on health and well-being can be evaluated by the incorporation of health within the EIA process or the development of separate legislative processes for Health Impact Assessment (HIA), which has been the case for a few countries.

HIA is a structured process and has several features that would enhance EIA, or as a separate approvals process, would predict and minimise adverse impacts and enhance the positive impacts of a development on human health and well-being. In this editorial we bring attention to certain features that we consider HIA can bring to the approvals system which are briefly discussed below.

## Public Participation

Active public participation in transparent decision-making processes can help promote inclusive, healthy and equitable communities. Democracy is a key value of HIA and engaging with communities during the planning stages of development activities can result in better health outcomes. 

Participation in the development and implementation of development projects, as well as policies and programs that may have an effect on their lives is important to communities and should be regarded as one of the most main components of HIA. People can have concerns about the potential for adverse effects on themselves, their families and communities. Similarly, people want to be involved in the decision-making process and have input into the developmental stages of new proposals. Addressing these, including the perception of potential concern, during engagement should provide greater community assurance. As identified by McNaughton *et al.*, there is the need for communities to be informed of their human rights with respect to health and the participation of disadvantaged and marginalized groups [4]. Additionally governments must be accountable to stakeholders on decisions made. James *et al.* emphasise the importance of stakeholder engagement even within rapid HIA [5].

Public participation can improve the effectiveness of an HIA through identification of community concerns, enabling people to contribute their knowledge, experiences and expertise and communities can provide potential support for the implementation by decision makers of recommendations arising from the HIA.

The terms public participation and community engagement are both used to describe the range of interactions between people. The options for working with communities can range from one-way communication or information delivery from the decision makers, direct consultation, involvement and collaboration in decision-making with community groups and individuals through to empowered action by informal groups or within formal partnerships.

Communities can be receptive to new proposals if the process is open and information about the proposal is provided in a form that is readily understood. Communities can benefit from the implementation of proposals, which aim for positive outcomes in health and wellbeing and reductions in social inequity. There is also potential for proponents to benefit from improved community interaction and recognition of their role as good corporate citizens.

However, it is important that any effects arising from decision-making are equitably distributed and lead to positive health outcomes for all members of communities. Central to this is the identification and empowerment of vulnerable populations. Gase *et al.* have outlined the difficulties that can occur when collaborating with multiple and diverse stakeholders when considering free public transit passes for students [6]. Korfmacher *et al.* have identified the need for multifaceted communication engagement programs for use in complex situations such as for waterways development [7].

Encouraging or requiring proponents and others to engage with communities will add depth to the health and wellbeing aspects of development proposals. It would be valuable for proponents to introduce the determinants of health to communities to identify issues not previously considered and determine issues regarded by the community as of significance. Not all the issues generated through participative processes may be included in the proposal however those with the potential to have significant impacts on the health and wellbeing of the community should definitely be included. Linzalone *et al.* considered the application of HIA within EIA and SEA in Italy and identified the value HIA methodologies would add to these processes [8].

It is important that community engagement is initiated as early as possible in the HIA process and that it continues as consultation and/or active participation throughout the developmental stages rather than at discrete and disconnected times within the assessment framework.

As well as being a means to provide transparency to the HIA process, community engagement methodology needs to demonstrate balance, rigour and reliability. There must be some means of being able to determine the level of community concern about issues and the degree to which these issues will impact on health. The community engagement methods chosen should ensure disadvantaged and other key stakeholder individuals and groups have access to adequate and appropriate information as well as having opportunities to provide information about issues specific to their needs and participate in decision-making processes where appropriate. Methods should be developed to address potential conflicts so that a level of understanding and agreement within the community can be reached and there should be appropriate means to communicate the outcomes of the HIA to the community.

Knoblauch *et al.* describe the processes used to consult communities in northern Sierra Leone [9]. Heller *et al.* found varying practices of community engagement within the policy development paradigm such as through HIAP and identified mechanisms in their HIA metrics to assist with addressing equity [10]. Similarly, Brown *et al.* provide advice on the processes used to address the needs of vulnerable groups and how to use the outcomes from consultation for adaptations to address health in climate change assessments [11].

## Health Risk Assessment

Risk is the chance of something happening that may have an impact on objects. All aspects of life involve exposure to risks but in all cases of threat, our priority is to evaluate the potential impacts on human health. 

Health risk assessment (HRA) is a tool for overall assessment and management of potential health threats from exposure to chemical, physical, biological and social hazards. It is considered to be a key component of the Health Impact Assessment (HIA) framework [12] and can be applied at the scoping and/or risk assessment stages but was not considered in most papers in this Special Issue “Health Impact Assessment”. 

Traditionally HIA used HRA to assess health consequences from exposures to environmental threats including air quality, noise, water quality, but overlooked a broader range of health determinants such as social determinants of health, for example culture and socioeconomic status. It is recommended that HRA is considered in future applications of the HIA framework and include all parameters that may have an impact on health risks. Consequences to health can be adverse but also beneficial such as those that arise from both social and environmental assets such as national parks, nature reserves, cultural areas and politics. A broader approach, taking into account environmental and social determinants and also both beneficial and adverse health impacts on people, should be considered in health risk assessment. The research paper by Knoblauch *et al.* is a good illustration for recognising and addressing health benefits related to a Bioenergy Project in Northern Sierra Leone [9]. 

HRA is a process of assessing the magnitude of risks associated with the hazards identified. This process requires combining consequences and likelihood estimates using a risk matrix that can provide different health risk levels ranging from low to extreme. It has been recommended that factors describing both acute and chronic exposures are extended and parameters associated with population size and potential health, economic costs and the perception/acceptance of the risks by the affected population [13] be included in the HRA process. This has been partially addressed in the paper by James *et al.* [5]. Similar proposals are raised in association with the likelihood ranking and the need for a better understanding of risk assessment uncertainties. These guidelines will enable those involved in HRA to have a more detailed description of the health impacts of a hazard beyond the usual fatality or injury models. This process is well addressed and demonstrated in both papers on climate change [11,14].

Hence, a more holistic approach in HIA is recommended in which all parameters that may influence risks are considered. This will provide a broader estimation of health consequences and a more consistent estimation of health risks at the HRA stage of the HIA process.

## Examples of Other Applications

HIA can be applied to ‘projects, plans or policies’ that traverse a wide range of situations and sectors including housing, transport, mining and agriculture. This necessitates a flexible framework that can incorporate sector-specific processes, tools or terminology relevant to the particular assessment. The incorporation of HIA principles into EIA is just one example of how HIA can facilitate more informed decision-making, particularly in relation to human health and well-being.

HIA principles can also been incorporated into the review or setting of air quality standards [15]. Air quality standard setting that is based on exposure and health outcome evidence for the general population can fail to adequately address issues of vulnerability and equity. For example, populations exposed to high levels of pollutants from localised sources may not be adequately protected by air quality standards. Similarly, problems may arise if sensitive population groups such as children or the chronically ill are not taken into account during standard setting. 

This special issue includes several papers that consider air quality as part of HIAs related to the transportation sector. James *et al*., identified reductions in air quality as one of the adverse health outcomes of proposed service cuts and fare increases, which ultimately influenced the decision to reduce the extent of the proposed fare increases and service cuts [5]. Comparably, while the HIA of a proposal to reduce speed limits concluded that air quality would worsen, it was determined the benefits of reduced road fatalities and injuries and higher levels of active transport outweighed the costs related to air quality and other factors [13]. Both these HIAs demonstrate the need to consider health outcomes in a systematic and holistic way and serve to highlight that decision-making must take a range of risks and benefits into account.

HIA has also been demonstrated as a useful framework for the issue of climate change [11,14]. The scope and complexity of the interactions between climate and health, as well as the long time frames and levels of uncertainty, present a considerable challenge when planning appropriate adaptation measures. The use of a HIA framework, augmented by key climate change terminology, concepts and methods, can provide much needed support for tackling this issue. For example, the systematic approach of HIA can be combined with evidence from key sources such as the Intergovernmental Panel on Climate Change (IPCC) to ensure issues of vulnerability, inequity and stakeholder consultation are factored into planning to address health impacts of climate change [16].

## Summary

The incorporation of HIA principles with well-established approvals processes or as a stand-alone process can offer significant benefits, and provide the best opportunity to influence decision-making with respect to health and well-being. Improving knowledge and capacity in HIA amongst decision makers will bring value rather than the current perception of adding to complexity and burden in approvals processes.
